# Sex work-related homicides: Insights from the National Violent Death Reporting System, 2012–2020

**DOI:** 10.1111/1556-4029.15434

**Published:** 2023-11-20

**Authors:** Brenda L. Nguyen, Katherine A. Fowler, Carter J. Betz, Kara Tsukerman, Sharon G. Smith

**Affiliations:** 1 Division of Violence Prevention, Centers for Disease Control and Prevention, Atlanta, Georgia, USA; 2 Division of Violence Prevention, Centers for Disease Control and Prevention, Oak Ridge Institute for Science and Education, ORISE Fellow, Atlanta, Georgia, USA

**Keywords:** crime victims, homicides, sex trafficking, sex work

## Abstract

Homicide is a prevalent cause of death among sex workers, given their increased risk of violence due to proximity to criminal activities such as drug trade and human trafficking. This study analyzes homicide data from the National Violent Death Reporting System (NVDRS) covering 49 US states, the District of Columbia, and Puerto Rico from 2012 to 2020. Case inclusion criteria included: (1) manner of death of homicide, and (2) sex work-related circumstance. Descriptive analyses examined victim and injury characteristics, suspect information, and circumstances. The study identified 321 sex work-related homicides (54% female, 41% male, 6% transgender). Among female victims, 94% were sex workers, and 54% of their suspects were clients. Money conflicts (23%) and other crimes (30%), most often in progress, commonly precipitated homicides of female victims. Substance use problems were reported in 49% of female victims, with 25% of their suspected perpetrators reportedly using substances in the preceding hours. For male victims, 54% were clients and 9% were sex workers. Suspects in male homicides were primarily sex workers (34%) or individuals engaged in sex work-adjacent criminal activities (36%). Money conflicts (49%), other crimes (47%) most often in progress, and sex trafficking involvement (25%) commonly precipitated homicides with male victims. Transgender sex worker victims were mostly transfeminine (94%) and non-Hispanic black (89%). Money conflicts (78%) most commonly precipitated homicides among transgender sex worker victims. These findings can inform prevention strategies addressing underlying risk factors for persons involved in sex work.

## 1 | INTRODUCTION

Homicide is a prevalent cause of death among sex workers [1–3]. In 2004, the largest and most recent study recording the mortality rate of female sex workers in the United States (US) reported women involved in sex work were approximately 18 times more likely to be murdered than women of similar age and race from 1967 to 1999 [2]. It is estimated that 2.7% of female homicide victims in the US between 1982 and 2000 were sex workers [4]. Male sex workers also have an increased vulnerability to high levels of violence and harassment [5]. By the nature of their work, sex workers are both publicly visible to strangers seeking to engage in illicit activities, and often conduct these transactions in secluded areas; these conditions increase the likelihood of sex workers experiencing violence such as assault and murder [4, 6, 7]. Sex worker homicides are often perpetrated by strangers and in secluded parts of populated areas [3]. Most sex worker homicide victims are adults, however, victims under 18 years of age have also been documented. Sex workers under the age of 18 are considered victims of human trafficking, as they are unable to willingly engage in sex work, according to the US Department of Justice’s definition of human trafficking [8].

Sex work often overlaps within larger organized crime structures that profit from activities like the drug trade, sex trafficking, and gambling [9, 10]. A systematic review by Ottisova et al. [11] found that women trafficked for sexual exploitation experience higher levels of sexual and physical violence compared to non-trafficked sex workers. Researchers have reported that the number of recorded sex work-related homicides (including those of clients and sex traffickers) have steadily increased since the 1970s and spiked prominently during the late 1980s and early 1990s. They also found that sex workers were murdered primarily by clients, clients were murdered mainly by sex workers, and pimps (or person who manages and profits from the activities of sex workers) were murdered predominantly by other pimps [4, 6]. Moreover, while we have gained insights into the victim-suspect dynamics within sex work-related homicides, a more detailed exploration is essential. The motivations, circumstances, and patterns behind these killings remain limited.

Challenges to detecting sex work-related homicides have been reported in homicide data sources. The rate of homicide among sex workers is likely underestimated given that an accurate denominator is hard to obtain due to lack of knowledge of the true number of sex workers [12]. There are issues related to the failure to recognize victims as sex workers, difficulties in identifying victims due to advanced decomposition, and cases where sex workers are classified as missing persons because their remains have never been found [13]. Additionally, sex work-related homicides have been under-ascertained in most datasets due to the lag time often required for law enforcement to solve such crimes. Other reasons may include fears of incrimination, lack of public interest due to a negative perception of the victim, and lack of credible witnesses [6]. Prior findings show lags often exceeding a year between the dates of a sex worker homicide and the arrest of potential perpetrators [4].

The majority of prior research on sex work-related homicide in the US used survey data from small cohorts of sex workers, homicide data from specific cities and states, information obtained from media reports on homicides involving sex work, and/or data from the FBI’s Supplementary Homicide Reports (SHR) database [1–6, 13], which relies on voluntary participation by law enforcement agencies and may therefore be incomplete [14, 15] (documentation of SHR data is available at https://www.ojjdp.gov/ojstatbb/ezashr/). These datasets have limitations in that they are often untimely, may lack comprehensive reports describing the circumstances leading to the deaths, and may not identify other sex work-related roles, such as clients and sex traffickers. Access to such detailed information can be restricted or difficult to obtain possibly due to legal and privacy constraints or the illicit nature of sex work activities [6, 16]. Additionally, the definition of sex work-related incidents can vary across jurisdictions, further complicating the compilation and analysis of data in this area of research. As a result, previous research may not give the full picture of sex work-related homicides.

The purpose of this study is to conduct a descriptive analysis of sex work-related homicides using timely and robust data from the National Violent Death Reporting System (NVDRS) in order to describe the characteristics of victims, suspected perpetrators (here on “suspects”), the relation of the victims and suspects to sex work, the injury incident, and precipitating circumstances. This is the largest and most detailed analysis of sex work-related homicides in the US to date. By building upon the existing research, addressing its limitations, and offering new insights into the complexities of this issue, these findings can inform prevention strategies addressing underlying risk factors for persons involved with sex work.

## 2 | MATERIALS AND METHODS

### 2.1 | Data

We analyzed data from all states and jurisdictions participating in the Centers for Disease Control and Prevention’s (CDC) National Violent Death Reporting System (NVDRS) for 1 or more years for deaths occurring from 2012 to 2020 (49 US states, the District of Columbia, and Puerto Rico). In this analysis, 13 states/jurisdictions did not report any sex-work related homicide cases and therefore the results reflect findings from 36 states, the District of Columbia, and Puerto Rico. The NVDRS, described in detail elsewhere [17], is an active, state-based surveillance system that links data on violent deaths (including homicides) from death certificates, coroner or medical examiner reports, and law enforcement reports into a single incident record. Information is abstracted by state-level abstractors using standard coding guidance developed by the CDC. The NVDRS defines homicide as a death resulting from the intentional use of force or power, threatened or actual, against another person, group, or community (International Classification of Diseases, 10th Revision, cause-of-death codes X85-X99, Y00-Y09, Y87.1, U01, and U02).

NVDRS includes data on characteristics of victims and suspects, the weapon(s) or method used to inflict the fatal injury, toxicology of the victim(s), and circumstances identified as being related to the death (including sex work). Circumstances related to a violent death are defined as the precipitating events (i.e., preceding or impending) in relation to the victim that contributed to the infliction of a fatal injury. These events can include factors such as mental health conditions, substance use problems, relationship and life stressors, and criminal activity. Data on precipitating circumstances often originate from investigators’ interviews with informants who knew the decedent. In addition, NVDRS data abstractors write narrative descriptions about the incident based on reports from coroners/medical examiners and law enforcement officers. The narratives serve to summarize the events of the fatal incident and other pertinent information, including additional context around circumstances.

### 2.2 | Case definition

Cases were defined as victims in NVDRS who had a manner of death of homicide where “prostitution” or “prostitution was a crisis” were selected as a circumstance (*N* = 321). These victims were involved in incidents where prostitution (also known as sex work) or prostitution-related activities played a precipitating role leading to the victim’s death. “Prostitution was a crisis” circumstance indicated a current/acute engagement in prostitution-related activity (within 2weeks of death) contributed to the victim’s death; for example, the victim, or sex worker, had a fight with a pimp and threatened to leave the job before being killed. These prostitution circumstance variables were added to NVDRS starting in data year 2012; therefore, years 2012–2020 (the most recent data available at the time of this analysis) were used. [18].

### 2.3 | Narrative review

NVDRS narratives were reviewed to: (1) ensure the incident met the case definition of sex work-related, and (2) code new variables created for purposes of the study. Coding guidance was developed by study authors BN and KF and reviewed by the other authors. New variables were double-coded by alternating pairs of co-authors to establish inter-rater reliability. Disagreements were resolved by group discussion until consensus was reached. The new variables developed for the study were:

- Victim’s/Suspect’s relation to or involvement in sex work at time of death, as encompassed by the following categories:
  1. Sex worker: The victim or suspect was a sex worker/prostitute or had history of sex work.
    - Sex trafficking victim: Following the US Department of Justice’s definition of human trafficking [7], victims or suspects who were sex workers under the age of 18 were considered victims of sex trafficking.
  2. Client/Prospective client: The victim or suspect was a client of a sex worker, including prospective clients/persons who attempted to solicit another person to exchange goods/services/money for sex or someone posing as a client for purposes of robbery, sexual assault, and/or homicide.
  3. Human Trafficker/Pimp: The victim or suspect was involved in sex trafficking as a trafficker. This includes persons working in human trafficking criminal organizations and individual sex traffickers such as pimps.
  4. Other person engaged in sex work-adjacent criminal activity: The victim or suspect was a drug dealer, gang member, or other person engaging in illicit or illegal business with ties to sex work through these activities.
  5. Bystander/observer: The victim or suspect was a bystander or observer of sex work-related activity but had no direct involvement.
  6. Intimate partner of sex worker: The victim or suspect was the current or former boyfriend, girlfriend, fiancé(e), domestic partner, or spouse of a sex worker. If the victim or suspect was also trafficking the decedent, they were classified as “human trafficker/pimp” as this is their primary relationship to sex work.

Additional incident characteristics:

- Serial homicide: The victim was considered part of a series of any number of separate but related homicides thought to be perpetrated by the same suspect.
- Sex worker was transgender: If the victim or suspect was a sex worker, and if the coroner and/or law enforcement reports noted the suspect or victim self-identified as transgender, or a friend/family member indicated that the victim self-identified as transgender.
- Sex trafficking-related: Whether the incident was noted to involve human trafficking/sex trafficking (exploitation of one or more persons for commercial sex). This included any situations involving human trafficking organizations (organized networks that engage in the trafficking of people), individual traffickers (i.e., pimps), or sexual exploitation of underage persons.
- Money conflict: Whether the incident was related to robbery or another conflict over money or property (e.g., dispute over payment).

### 2.4 | Statistical analysis

We computed descriptive statistics to examine characteristics of the sex work-related homicide victims and suspects, precipitating circumstances, and other injury characteristics stratified by sex of the victim. Data for transgender sex workers were analyzed separately, as they identify different from their biological sex and may be impacted differently. Chi-square (*χ*^2^) analyses were used to test for associations between male/female victims and demographic and injury characteristics. We did not perform chi-square tests for transgender sex workers because of their small sample size. Analyses were considered statistically significant based on *p* ≤ 0.05. We used SAS Version 9.4 (SAS Institute) for all analyses.

## 3 | RESULTS

### 3.1 | Victims of sex work-related homicide

A total of *N* = 303 victims of sex work-related homicides, excluding transgender victims (described in detail later), were recorded in NVDRS between 2012 and 2020 (Table 1). Fifty-seven percent (*N* = 172) were female, 43% (*N* = 131) were male. Ten of these homicides (3.3%; 4 female victims and 6 male victims) were reportedly known to be part of a series of homicides (i.e., serial killings; data not shown); none were found to be interconnected. There were significant differences in victim characteristics by sex. Female victims were more likely younger, single, and had lower levels of college education compared to male victims. Female victims were often sex workers and non-Hispanic white, while more than half of male victims were clients and often non-Hispanic black. Both groups were predominantly injured by firearms, with a higher percentage among male victims. The relationship of victim to suspect varied significantly by sex. Female victims were more likely to be killed by a current or former spouse or intimate partner compared to male victims, while male victims were more likely killed by strangers compared to female victims.

### 3.2 | Female victims of sex work-related homicide

#### 3.2.1 | Demographic characteristics

Ninety-four percent of female victims of sex work-related homicides were sex workers; an additional 2% were underage victims of sex trafficking (Table 1). The largest percentage of female victims were aged 25–34 years (32%). The majority of female victims were non-Hispanic white (54%); 38% were non-Hispanic black and 6% were Hispanic of any race. Seventy-one percent of female victims were single/never married, and most had a high school education (47%) or less (29%).

#### 3.2.2 | Injury characteristics

Forty-one percent of female victims were killed by firearm injuries (Table 1). Twenty-one percent died by strangulation/suffocation/hanging, and 19% died from sharp instrument wounds. Each of the other methods represented <10% of fatal injuries. Thirty-eight percent of sex work-related female homicide victims were killed in a house/apartment (48% of these were in the victim’s own home); 16% were killed on a street/road/sidewalk/alley and 15% were killed in a hotel/motel. Other locations of injury (motor vehicle, natural area, and other locations) represent 10% or less each. Seventeen percent of female victims were killed by a current or former intimate partner; 44% of which were also involved in trafficking the victim (data not shown).

### 3.3 | Male victims of sex work-related homicide

#### 3.3.1 | Demographic characteristics

Fifty-four percent of male victims of sex work-related homicides were clients; 9% were sex workers (Table 1). More than one in five male victims engaged in sex work-related criminal activities including human trafficking (17%) or other sex work-adjacent crime (8%). Most male victims were non-Hispanic black (53%); 34% were non-Hispanic white and 12% were Hispanic. Fifty-seven percent of male victims were single/never married. Most victims had a high school education (46%) or less (22%).

#### 3.3.2 | Injury characteristics

Sixty-six percent of male victims were killed by firearm injuries and 18% died from sharp instrument wounds (Table 1). Each of the other methods represented <10% of fatal injuries. Forty-six percent of sex work-related homicides with male victims occurred in a house/apartment (73% of these in the victim’s own home). Fifteen percent were killed in a hotel/motel, 14% were killed on a street/road/sidewalk/alley, and 11% in a motor vehicle. Other locations of injury (natural area, parking lot/public garage, abandoned house/building, and other locations) represent 8% or less each.

### 3.4 | Suspect characteristics

Characteristics of a total of *N* = 322 suspects were involved in sex work-related homicides (*N* = 150 suspects in female-victim incidents; *N* = 172 in male-victim incidents; Table 2). Some incidents had multiple suspects involved, in addition to the primary suspect responsible for the victim’s death; Table 2 represents all suspects in sex work-related homicides. There were several statistically significant differences between suspect characteristics and victim sex. Suspects of female victims were more often clients, male, younger (under 34years of age), had a suspected history of substance use in the hours preceding the homicide, a history of abusing the victim, and a higher likelihood of attempted suicide following the victim’s death. Out of the incidents with informative (i.e., not missing or unknown) suspect data (*N* = 252), 22.6% of incidents had more than one suspect, ranging from two to five suspects (data not shown).

#### 3.4.1 | Incidents with female victims

Ninety-two percent of suspects with female victims were male (Table 2). The majority were aged 18–24 years (21%) and 25–34 years (29%). Most were non-Hispanic black (51%); 24% were non-Hispanic white. Fifty-four percent of suspects of female victims were a client of the victim; an additional 18% were a human trafficker/pimp, and 15% were involved in other sex work-adjacent criminal activity. Twenty-five percent of suspects in incidents with female victims reportedly had used substances (not including alcohol) in the hours prior to the incident; 12% had contact with law enforcement in the 12 months prior.

#### 3.4.2 | Incidents with male victims

Sixty-nine percent of suspects with male victims were male and 28% were female (Table 2). The majority were aged 18–24 (30%) and 25–34years (32%). Approximately half were non-Hispanic black (54%); 32% were non-Hispanic white. Thirty-six percent of suspects in incidents with male victims were a person involved in other sex work-adjacent criminal activity; 34% were sex workers; and 12% were clients. Six percent of suspects in incidents with male victims reportedly had used substances in the hours prior to the incident; 15% had contact with law enforcement in the 12 months prior; and 9% had been released from an institutional setting (e.g., jail, prison, inpatient psychiatric care) in the month prior to the incident.

### 3.5 | Precipitating circumstances

Table 3 describes the precipitating circumstances of sex work-related homicide victims. Male victims were more likely to be involved in money conflicts and sex trafficking, as well as to have alcohol-related problems compared to female victims. Female victims were more likely to have substance use problems and to experience intimate partner violence compared to male victims. Most often, homicides were precipitated by another crime among male victims, with a higher likelihood of robbery and involvement in the drug trade compared to female victims, whereas female victims were more likely to experience fatal or nonfatal assault, rape, or sexual assault compared to male victims.

#### 3.5.1 | Incidents with female victims

The most common circumstances leading up to sex work-related homicide with female victims were related to crimes other than the fatal assault (30%), most often in progress at the time of the incident (63%; Table 3). Most commonly the crime in progress was robbery (39%), rape/sexual assault (28%), or assault/homicide (26%). Twenty-three percent of incidents were precipitated by money conflicts (e.g., robbery, dispute over payment); 19% were related to drug involvement (e.g., relating to ongoing substance use or illicit drug trafficking); and 13% were sex trafficking related. Other common precipitating circumstances included an argument or conflict (22%) or being intimate partner violence-related (16%). Forty-nine percent of female victims of sex work-related homicide had noted substance use problems (excluding alcohol).

#### 3.5.2 | Incidents with male victims

The most common circumstances leading up to sex work-related homicide with male victims were money conflicts (49%); being precipitated by another crime (47%), most often in progress at the time of the incident (79%); arguments (23%); and/or a physical fight between two people (16%, Table 3). Most often the crime in progress was robbery (69%) or assault/homicide (18%). Twenty-five percent were sex trafficking-related and 20% were related to drug involvement. Twenty-eight percent of male victims of sex work-related homicide had noted substance use problems (excluding alcohol).

### 3.6 | Transgender sex worker victims

#### 3.6.1 | Demographics

A total of *n* = 18 (9.4%) sex worker victims were transgender. Of these, 94% were transfeminine (a person assigned male at birth who identified as female), and most were non-Hispanic black (89%) followed by Hispanic (11%) (data not shown). Sixty-seven percent of transgender victims were killed by firearm injuries and 22% died from sharp instruments. Forty-four percent were killed in a house/apartment (22% of these occurring in the victim’s own home) and 22% were killed on street/road/sidewalk/alley.

#### 3.6.2 | Suspect characteristics and precipitating circumstances

Among transgender victims, suspect relation to or involvement in sex work was unknown for half the suspects (50%) (data not shown). Of suspects with a known relation to or involvement in sex work (*N* = 9), most were a client of the victim (89%). The most common circumstances leading up to homicide with transgender sex worker victims were money conflicts (78%); being precipitated by another crime (22%); arguments (22%); and/or a physical fight between two people (13%). Seventeen percent of transgender victims of sex work-related homicide had noted substance use problems (excluding alcohol).

## 4 | DISCUSSION

Sex work-related homicides is a public health concern, and it is important to understand the factors contributing to these incidents. In this study, we analyzed NVDRS data from 2012 to 2020 to conduct a detailed analysis of the demographics, injury characteristics, suspect characteristics, and precipitating circumstances surrounding sex work-related homicides in the US. Our findings reveal disparities among victims based on sex and provide an understanding of experiences of transgender victims, which provides insights into the unique challenges faced by these groups. These findings can inform prevention strategies addressing underlying risk factors for persons involved in sex work.

Approximately half of male victims were clients, the vast majority of female victims were sex workers. Perpetrators of female victims’ homicides were most often clients, which has been noted in past studies [4, 19, 20]. Unlike their female counterparts, male victims were more likely to be killed by individuals who engage in sex work-adjacent criminal activity including sex traffickers or by sex workers. Our study revealed that more than half of the victims were injured in a house/apartment/rooming house or hotel/motel. In contrast, prior research stated that sex workers are particularly vulnerable to homicide due to working and providing services in secluded areas [3, 6]. Among female victims who were injured in a house/apartment/rooming house, almost half were injured in their own homes. Among male victims injured in the same setting, almost three-quarters were injured in their own homes. In cases of home injuries, victims may be involved in various risky situations, such as conducting sex work at their own residence, encountering stalkers, being robbed, or having high-risk encounters with individuals involved in illicit activities at their residence. Tailored strategies are important to address the distinct risks faced by male and female victims within the sex work industry, providing resources for harm reduction and support services for victims facing harm such as stalking and other acts of violence.

Almost half of female victims had noted substance use problems. The relationship between drug use and sex work among women in the sex industry has been previously studied [21–23]. Substance use among sex workers is often used as a coping mechanism for psychological distress attributed to the stigma and discrimination they face [21]. Substances are a common tool of recruitment and control utilized by traffickers. Some seek victims that struggle with substance use disorders, and others introduce highly addictive substances, often without the victim’s knowledge, as a way to ease them into the work, reward for productivity, and exert power and punishment by initiating withdrawal symptoms [22, 23]. Stigma is often a major barrier for women involved in sex work and substance use, and access to harm reduction services is often limited or non-existent. Harm reduction strategies and policy changes can help address the intersecting issues of stigma, sex work, and substance use [24].

Money conflicts were a common circumstance among both female and male victims. This could be due to clients’ preference for cash payments to maintain anonymity which can lead to an increased risk of robbery and assault due to knowledge of high cash flow in sex work business and a reluctance to report theft to the authorities [25]. Additionally, clients may target on sex workers’ inability to report lack of payment of services to authorities due to the illicit nature of the transaction. Sex workers in these cases may have deterred from seeking help or reporting crimes, given their fears of incrimination and the lack of credible witnesses [6]. Therefore, interventions are needed to address these barriers and improve access to support and protection.

This study identified a total of 5 minor sex trafficking victims including one male victim. The prevalence of sex trafficking is likely underestimated because of its hidden nature, lack of law enforcement training to recognize victims, and lack of standardization in measurement [26, 27]. Furthermore, males are less likely to be recognized as victims of sex trafficking or report their own victimization [28, 29] due to pervasive gender stereotypes of female vulnerability and male masculinity, male sexual insatiability, and the stigma associated with male victimization [30]. Sex trafficking victimization of both females and males can be influenced by a range of risk factors, including poverty, social isolation, and a history of abuse or neglect [31]. Stigma and criminalization of sex work can also increase vulnerability to trafficking [32]. Addressing these underlying factors through harm reduction approaches and improved access to healthcare, social services, and legal protections can help prevent sex trafficking and reduce the harm caused by exploitation [33]. Sex trafficking is preventable through strategies such as increasing community awareness of human trafficking [34] and addressing exploitation after it occurs. Evidence-based strategies to prevent related forms of violence may also reduce sex trafficking and other violence against sex workers. These include strategies that encourage healthy relationship behaviors, promote safe homes and neighborhoods, provide opportunities to identify and address vulnerabilities during health care visits, reduce the commercial sex demand [35], and stop businesses from profiting from trafficking. Further, empowering women and girls through education, employment and community involvement are important strategies to reduce risk for sexual violence, including violence related to sex work and trafficking [36]. Gender inequality in these domains has been found to increase risk for sexual violence and to create conditions that may lead to engaging in sex work out of financial necessity [37]. Policies and programs that improve economic conditions and provide opportunities for women and girls can reduce this risk and other associated risks, and may include policies addressing income equality, access to affordable quality childcare, and leadership programs for young women and girls [36].

Our findings provide new insights on the incidents surrounding transgender sex worker homicide victims. The majority of the transgender sex worker victims were transfeminine (a person assigned male at birth who identified as female) and non-Hispanic black. This observation aligns with existing reports demonstrating a trend where fatal violence against transgender people disproportionately affects people of color, with black transfeminine individuals accounting for a plurality [38, 39]. Our findings showed transgender victims had a high prevalence of firearm-related fatalities. The precipitating circumstances leading to homicides among transgender individuals were often linked to money conflicts and involvement in other crimes. Transgender persons are known to be at higher risk for engaging in sex work and sex trafficking victimization, including survival sex while experiencing homelessness, and experiencing high levels of violence while engaging in sex work [5, 40, 41]. This may stem from multiple structural challenges related to transphobia [42], including exclusion from traditional forms of employment, lack of access to livable wages, safe and affordable housing, and lack of availability of trans-friendly service providers [40, 43, 44, 45]. Our findings emphasize the need for research to include victims assigned male at birth, particularly to study homicides among transfeminine sex workers, as previous research primarily focused on female victims while excluding male victims [1, 2, 6, 12, 20]. Further research is essential to better address the social marginalization and violence experienced by transgender sex workers, considering the needs of diverse racial and ethnic groups within the transgender community.

### 4.1 | Strengths

This study represents the largest analysis to date of sex work-related homicides in the United States. Many past studies have used limited or state-specific data sources, include older data, and/or have solely focused on homicides of sex workers but not others involved in the sex trade [2, 4, 12]. Most homicide datasets have limited information on the precipitating circumstances of incidents; however, this study used NVDRS data which contains comprehensive information from multiple sources including death certificates, law enforcement and coroner/medical examiner reports. This enables a more thorough investigation of the details surrounding sex work-related homicides.

### 4.2 | Limitations

This study has some limitations. First, NVDRS data were not nationally representative at the time of this report, and not all states joined the system at the same time. Second, the completeness of the data relies on strong partnerships among VDRS programs and their partners in vital records, medical examiner/coroner offices, and law enforcement, so the availability and completeness of data may vary in areas where these relationships are not fully developed. Abstractors are limited to the information they receive, and some VDRS programs do not receive detailed law enforcement reports until cases have been adjudicated. Third, NVDRS did not introduce the sex work-related circumstance (a.k.a., “prostitution”) variables until data year 2012, limiting the number of data years available for analysis. Fourth, NVDRS does not always receive detailed reports about motivations of suspect due to reasons such as ongoing investigations, privacy concerns, or lack of witnesses to the incident. In our study, we were unable to determine the relation to sex work for approximately 25% of suspects with informative data, and as a result, they were excluded in the percentages reported for suspects with a known relationship to sex work. Lastly, NVDRS narratives vary in the amount of detail provided by coroners and law enforcement. Certain information, including variables coded from the narrative data for this study, may have been under-captured due to a lack of details documented in the narratives. This study presents data on trafficked victims below the age of 18. However, sex workers aged 18years and older were not classified as trafficked victims or non-victims due to the challenge of clearly distinguishing between sex workers who were trafficked and those who engaged in sex work by choice within the narrative data. However, we noted if the incident was sex-trafficking related if it was indicated in the narratives. Additionally, transgender status is not consistently recorded in routine data collection, and victims can be misgendered by death investigators, which can result in an underestimation of the prevalence of transgender individuals within the NVDRS data.

## 5 | CONCLUSION

The majority of sex work-related homicide victims were female sex workers. Prevention strategies promoting economic opportunities and financial security for women and girls, safe and healthy relationships and environments, treatment for substance use disorders, and recognition and prevention of sex trafficking may reduce homicides of sex workers and others involved with the sex trade. Future research should examine the possibility of unique risk factors and prevention strategies among subgroups such as unhoused, transgender, and trafficking victims.

## Figures and Tables

**TABLE 1:** Characteristics of victims of sex work-related homicides by sex—National Violent Death Reporting System, 36 US states^a^, the District of Columbia, and Puerto Rico, 2012–2020^b^.

	Victim			
Characteristic	Female No. (%)	Male No. (%)	Total No. (%)	*p*-Value
Age group (years)				<0.001
<18	4 (2.3)	1 (<1.0)	5 (1.7)	
18–24	41 (23.8)	13 (9.9)	54 (17.8)	
25–34	55 (32.0)	39 (29.8)	94 (31.0)	
35–44	35 (20.3)	23 (17.6)	58 (19.1)	
45–54	32 (18.6)	27 (20.6)	59 (19.5)	
≥55	5 (2.9)	28 (21.4)	33 (10.9)	

Race/Ethnicity				0.0029
White, non-Hispanic	92 (53.5)	44 (33.6)	136 (44.9)	
Black, non-Hispanic	65 (37.8)	69 (52.7)	134 (44.2)	
American Indian/Alaska Native, non-Hispanic	3 (1.7)	0 (0.0)	3 (<1.0)	
Asian/Pacific Islander	1 (<1.0)	2 (1.5)	3 (<1.0)	
Hispanic^c^	11 (6.4)	16 (12.2)	27 (8.9)	

Marital status				0.0073
Married	10 (5.8)	18 (13.7)	28 (9.2)	
Single or never married	122 (70.9)	74 (56.5)	196 (64.7)	
Widowed	5 (2.9)	0 (0.0)	5 (1.7)	
Divorced or separated	30 (17.4)	34 (26.0)	64 (21.1)	
Unknown	5 (2.9)	5 (3.8)	10 (3.3)	

Education				0.016
Less than high school graduate or GED certificate equivalent	50 (29.1)	29 (22.1)	79 (26.1)	
High school graduate or GED certificate equivalent	80 (46.5)	60 (45.8)	140 (46.2)	
Some college or more	20 (11.6)	32 (24.4)	52 (17.2)	
Unknown	22 (12.8)	10 (7.6)	32 (10.6)	

Victim’s relation to or involvement in sex work				<0.001
Sex worker	161 (93.6)	12 (9.2)	173 (57.1)	
Client	0 (0.0)	71 (54.2)	71 (23.4)	
Human trafficker/pimp	0 (0.0)	22 (16.8)	22 (7.3)	
Other person engaged in sex work-adjacent criminal activity	1 (<1.0)	10 (7.6)	11 (3.6)	
Bystander/observer	1 (<1.0)	10 (7.6)	11 (3.6)	
Sex trafficking victim (<18 years of age)	4 (2.3)	1 (<1.0)	5 (1.7)	
Unknown	5 (2.9)	5 (3.8)	10 (3.3)	

Method				<0.001
Firearm	70 (40.7)	87 (66.4)	157 (51.8)	
Sharp instrument	32 (18.6)	23 (17.6)	55 (18.2)	
Hanging/Strangulation/Suffocation	36 (20.9)	9 (6.9)	45 (14.9)	
Blunt instrument	15 (8.7)	4 (3.1)	19 (6.3)	
Personal weapons (e.g., hands, feet, fists)	8 (4.7)	4 (3.1)	12 (4.0)	
Other method^d^	10 (5.8)	3 (2.3)	13 (4.3)	
Unknown	1 (<1.0)	1 (<1.0)	2 (<1.0)	

Location of injury				0.52
House/Apartment/Rooming house^e^	65 (37.8)	60 (45.8)	125 (41.3)	
Injury occurred at victim’s home^f^	31 (47.7)	44 (73.3)	75 (60.0)	0.014^f^
Hotel/Motel	26 (15.1)	20 (15.3)	46 (15.2)	
Street/road, sidewalk/alley, highway/freeway	27 (15.7)	18 (13.7)	45 (14.9)	
Motor vehicle	15 (8.7)	14 (10.7)	29 (9.6)	
Natural area	13 (7.6)	4 (3.1)	17 (5.6)	
Other location^g^	18 (10.5)	11 (8.4)	29 (9.6)	
Unknown	8 (4.7)	4 (3.1)	12 (4.0)	

Relationship of victim to suspect^h^				0.002
Acquaintance/Friend	35 (33.0)	30 (33.0)	65 (33.0)	
Other person, known to victim	31 (29.2)	26 (28.6)	57 (28.9)	
Stranger	22 (20.8)	33 (36.3)	55 (27.9)	
Spouse/Intimate partner (current or former)	18 (17.0)	2 (2.2)	20 (10.2)	

Total	172 (100)	131 (100)	303 (100)	

*Note*: Transgender sex workers were examined as a separate subgroup (data not shown due to low cell counts).

Abbreviation: GED, general equivalency diploma.

a US states include Alabama, Alaska, Arizona, California, Colorado, Connecticut, Delaware, Georgia, Illinois, Indiana, Iowa, Kansas, Kentucky, Louisiana, Maine, Maryland, Massachusetts, Michigan, Missouri, Nevada, New Jersey, New Mexico, New York, North Carolina, Ohio, Oklahoma, Oregon, Pennsylvania, Rhode Island, South Carolina, Tennessee, Texas, Virginia, Washington, West Virginia, and Wisconsin.

b Percentages might not total 100% due to rounding.

c Includes persons of any race.

d Other method includes (in descending order): motor vehicles (e.g., buses, motorcycles, other transport vehicles), poisoning, fall, fire/burns, intentional neglect, other (e.g., taser, electrocution, nail gun, intentional neglect, personal weapons).

e Includes both the building itself and the area directly outside, such as a driveway, porch, front walk, or garage.

f Denominator is decedents whose location of injury was House/Apartment/Rooming house.

g Other location includes (in descending order): parking lot/public garage/public transport, abandoned house/building/warehouse, commercial/retail area, park/playground/sports or athletic area, office building, industrial or construction area, preschool/school/college/ school bus, farm, and other unspecified location.

h Denominator is the number of homicide decedents with a known victim-to-suspect relationship (*n* = 197 [65.0%]; 91 [69.5%] males and 106 [61.6%] females); victim-to-suspect relationship was unknown for 106 decedents. The following sentence can be used as a guide for interpreting victim-suspect relationship: “The victim is the ____________ of the suspect”. For example, when a parent kills a child, the relationship is “Child” not “Parent” (“The victim is the child of the suspect”). Please note that this sentence is intended to be a general guide. However, some relationships may not be captured by this sentence (e.g., other person known to victim).

**TABLE 2:** Characteristics of suspects of sex work-related homicides by victim sex—National Violent Death Reporting System, 36 US states^a^, the District of Columbia, and Puerto Rico, 2012–2020^b^.

	Victim			
Characteristic	Female No. (%)	Male No. (%)	Total No. (%)	*p*-Value
Suspect sex				<0.001
Male	138 (92.0)	119 (69.2)	257 (79.8)	
Female	8 (5.3)	48 (27.9)	56 (17.4)	
Unknown	4 (2.7)	5 (2.9)	9 (2.8)	

Suspect age group (years)				0.0077
<18	4 (2.7)	9 (5.2)	13 (4.0)	
18–24	31 (20.7)	52 (30.2)	83 (25.8)	
25–34	43 (28.7)	55 (32.0)	98 (30.4)	
35–44	24 (16.0)	26 (15.1)	50 (15.5)	
45–54	10 (6.7)	4 (2.3)	14 (4.3)	
≥55	10 (6.7)	1 (<1.0)	11 (3.4)	
Unknown	28 (18.7)	25 (14.5)	53 (16.5)	

Suspect race/ethnicity				0.096
White, non-Hispanic	36 (24.0)	55 (32.0)	91 (28.3)	
Black, non-Hispanic	77 (51.3)	93 (54.1)	170 (52.8)	
American Indian/Alaska Native, non-Hispanic	1 (<1.0)	2 (1.2)	3 (<1.0)	
Asian/Pacific Islander, non-Hispanic	3 (2.0)	0 (0.0)	3 (<1.0)	
Hispanic^c^	12 (8.0)	7 (4.1)	19 (5.9)	
Other, non-Hispanic	17 (11.3)	14 (8.1)	31 (9.6)	
Unknown race, non-Hispanic	4 (2.7)	1 (<1.0)	5 (1.6)	

Suspect’s relation to or involvement in sex work^d^				<0.001
Client	60 (54.1)	17 (12.2)	77 (30.8)	
Other person engaged in sex work-adjacent criminal activity	17 (15.3)	50 (36.0)	67 (26.8)	
Sex worker	2 (1.8)	47 (33.8)	49 (19.6)	
Human trafficker/pimp	20 (18.0)	14 (10.1)	34 (13.6)	
Intimate partner of sex worker (current or former)	12 (10.8)	3 (2.2)	15 (6.0)	
Bystander/observer	0 (0.0)	5 (3.6)	5 (2.0)	
Sex trafficking victim (<18 years of age)	0 (0.0)	3 (2.2)	3 (1.2)	

Other suspect characteristics				
Suspected substance use by the suspect in the hours preceding the incident^e^	24 (24.5)	7 (5.6)	31 (13.9)	<0.001
Suspect had contact with law enforcement in the past 12 months^e^	12 (12.2)	19 (15.2)	31 (13.9)	0.53
Suspect injured victim within a month of being released from or admitted to an institutional setting^e^	4 (4.1)	11 (8.8)	15 (6.7)	0.16
Suspected alcohol use by the suspect in the hours preceding the incident^e^	7 (7.1)	5 (4.0)	12 (5.4)	0.30
History of abuse of victim by the suspect	8 (5.3)	0 (0.0)	8 (2.5)	0.0022
Suspect’s attack on the victim is believed to be the direct result of a mental illness	5 (3.3)	2 (1.2)	7 (2.2)	0.18
Suspect attempted suicide (fatally or non-fatally) after the death of the victim	4 (2.7)	0 (0.0)	4 (1.2)	0.031
Suspect for this victim was also a victim in the incident (i.e., a victim/suspect)^f^	2 (1.5)	2 (1.2)	4 (1.3)	0.83
Suspect was a caregiver for the victim	2 (1.3)	0 (0.0)	2 (<1.0)	0.13

Total	150 (100)	172 (100)	322 (100)	

*Note*: Transgender sex workers were examined as a separate subgroup (data not shown due to low cell counts).

a US states include Alabama, Alaska, Arizona, California, Colorado, Connecticut, Delaware, Georgia, Illinois, Indiana, Iowa, Kansas, Kentucky, Louisiana, Maine, Maryland, Massachusetts, Michigan, Missouri, Nevada, New Jersey, New Mexico, New York, North Carolina, Ohio, Oklahoma, Oregon, Pennsylvania, Rhode Island, South Carolina, Tennessee, Texas, Virginia, Washington, West Virginia, and Wisconsin.

b Percentages might not total 100% due to rounding. Denominator is the total number of suspects with informative data (i.e., not missing or unknown). Some victims had >1 suspect. Of the 303 total victims (see Table 1—172 females, 131 males), 258 (85.1%) had informative suspect data. Females: 136 of 172 victims (79.1%) and males: 122 of 131 (93.1%) had informative suspect data. Those 136 female victims had 150 total suspects (1.10 suspects per victim) and the 122 male victims had 172 total suspects (1.41 suspects per victim).

c Includes persons of any race.

d Denominator is suspects with a known relation to or involvement in sex work (*n* = 250 [77.6%]; 111 [74.0%] females and 139 [80.8%] males); suspect relation to or involvement in sex work was unknown for 72 suspects.

e Variable began data collection in 2016. Denominator is sex work-related homicides with informative suspect data, 2016–2020 (*n* = 223; 125 males and 98 females).

f Variable began data collection in 2013. Denominator is sex work-related homicides with informative suspect data, 2013–2020 (*n* = 307; 170 males and 137 females).

**TABLE 3:** Number^a^ and percentage^b^ of sex work-related homicides by victim sex and precipitating circumstances^c^ – National Violent Death Reporting System, 36 US states^d^, the District of Columbia, and Puerto Rico, 2012–2020.

	Victim			
Precipitating circumstances	Female No. (%)	Male No. (%)	Total No. (%)	*p*-Value
Sex work-related				
Money conflict (including robbery)	39 (22.7)	64 (48.9)	103 (34.0)	<0.001
Sex trafficking	22 (12.8)	33 (25.2)	55 (18.2)	0.0055

Crime related				
Precipitated by another crime^e^	51 (29.7)	61 (46.6)	112 (37.0)	0.0025
Robbery	20 (39.2)	42 (68.9)	62 (55.4)	<0.001
Assault/Homicide	13 (25.5)	11 (18.0)	24 (21.4)	0.0097
Rape/Sexual assault	14 (27.5)	1 (1.6)	15 (13.4)	<0.001
Drug trade	2 (3.9)	6 (9.8)	8 (7.1)	0.0071
Other crime^f^	9 (17.6)	16 (26.2)	25 (22.3)	0.0084

Drug involvement	32 (18.6)	26 (19.8)	58 (19.1)	0.79

Gang-related	5 (2.9)	7 (5.3)	12 (4.0)	0.28

Walk by assault^g^	3 (1.9)	4 (3.1)	7 (2.4)	0.48

Mental health or substance abuse				
Substance abuse problem (excludes alcohol)	84 (48.8)	36 (27.5)	120 (39.6)	<0.001
Current diagnosed mental health problem	13 (7.6)	5 (3.8)	18 (5.9)	0.17
Alcohol problem	5 (2.9)	11 (8.4)	16 (5.3)	0.034
History of ever being treated for a mental health problem	10 (5.8)	3 (2.3)	13 (4.3)	0.13
Current mental health or substance abuse treatment	6 (3.5)	1 (<1.0)	7 (2.3)	0.12

Relationship and life stressors				
Argument or conflict	38 (22.1)	30 (22.9)	68 (22.4)	0.87
Physical fight between 2 individuals^g^	14 (8.6)	20 (15.6)	34 (11.7)	0.066
Intimate partner violence-related	28 (16.3)	4 (3.1)	32 (10.6)	<0.001
Problem with a friend or associate	9 (5.2)	11 (8.4)	20 (6.6)	0.27
Problem with a family member (not intimate partner)	4 (2.3)	1 (<1.0)	5 (1.7)	0.29

Total	172 (100)	131 (100)	303 (100)	

*Note*: Transgender sex workers were examined as a separate subgroup (data not shown due to low cell counts).

a All 303 sex work-related homicide records included one or more precipitating circumstances. Total numbers do not equal the sums of the columns because more than one circumstance could have been present per decedent.

b The sums of percentages in columns exceed 100% because more than one circumstance could have been present per decedent.

c Circumstance data elements in NVDRS are part of the victim’s record and apply to victims in an incident.

d US states include Alabama, Alaska, Arizona, California, Colorado, Connecticut, Delaware, Georgia, Illinois, Indiana, Iowa, Kansas, Kentucky, Louisiana, Maine, Maryland, Massachusetts, Michigan, Missouri, Nevada, New Jersey, New Mexico, New York, North Carolina, Ohio, Oklahoma, Oregon, Pennsylvania, Rhode Island, South Carolina, Tennessee, Texas, Virginia, Washington, West Virginia, and Wisconsin.

e Most homicides that were precipitated by another crime occurred while the other crime was in progress (*n* = 80 [71.4%]; 32 [62.7%] females and 48 [78.7%] males). Denominators for the list of other crimes that precipitated the homicide are the respective female, male, and total counts in the row ‘Precipitated by another crime.’ Percentages for the other crimes do not sum to 100% because a decedent could have experienced more than one other crime.

f Other crime includes (in descending order): burglary, motor vehicle theft, arson, witness intimidation/elimination, other unspecified crime.

g Variable began data collection in 2013. Denominator is sex work-related homicides with informative suspect data, 2013–2020 (*n* = 290; 128 males and 162 females).

## Highlights

- This study examined sex work-related homicides in the U.S. occurring between 2012 and 2020.
- The research explores the characteristics of victims, suspects, and their relation to sex work.
- We examined precipitating circumstances (e.g., trafficking, money conflicts, substance use).
- Money conflicts were a common precipitating circumstance among both female and male victims.
- Economic opportunities and substance use treatment can reduce sex work-related homicides.
